# Long non-coding RNA ZFAS1 regulates cell proliferation and invasion in cervical cancer via the miR-190a-3p/KLF6 axis

**DOI:** 10.1080/21655979.2021.2022265

**Published:** 2022-02-03

**Authors:** Yuehui Su, Wenjing Hou, Chunyan Zhang, Pengcheng Ji, Rui Hu, Qiongying Zhang, Yao Wang, Panpan Li, Huiping Zhang, Yueyue Chen, Xiaodong Zhang, Mengzhen Zhang

**Affiliations:** Department of Gynaecology, The First Affiliated Hospital of Zhengzhou University, Zhengzhou, PR. China

**Keywords:** ZFAS1, miR-190a-3p, KLF6, cervical cancer

## Abstract

Long non-coding RNA (lncRNA) ZFAS1 (zinc finger antisense 1) was demonstrated to play critical roles in various cancer progression. However, the functions of ZFAS in cervical cancers (CC) are unclear. Human CC cell lines were used for *in vitro* experiments. RT-qPCR (Real Time Quantitative PCR) was performed to detect the expression of ZFAS1, microRNA-190a-3p (miR-190a-3p) and Kruppel-like factor 6 (KLF6). Cell proliferation, invasion and migration assays were used to investigate biological behaviors of CC cells related to CC progression. The relationship of KLF6 to ZFAS1 and miR-190a-3p was analyzed by circRIP and luciferase reporter assay. In addition, *in vivo* experiment was carried out to explore the function of ZFAS1 in tumor growth of CC. The expression levels of ZFAS1 and KLF6 were both significantly elevated, while the expression of miR-190a-3p was inhibited in CC tumor tissues. In addition, ZFAS1 influenced CC tumor growth through miR-190a-3p. KLF6 was a target of miR-190a-3p and inhibited miR-190a-3p-induced CC tumor growth. Furthermore, KLF6 was negatively regulated by miR-190a-3p, but positively regulated by ZFAS1. Overexpression of ZFAS1 and inhibition of miR-190a-3p significantly increased the expression levels of KLF6. Finally, *in vitro* assays demonstrated that inhibition of ZFAS1 reduced CC tumor growth and the expression levels of KLF6, but increased the expression levels of miR-190a-3p. ZFAS1 could regulate CC pathogenesis via regulating the miR-190a-3p/KLF6 axis, which might be considered as new CC therapeutic targets.

## Introduction

Nowadays, cervical cancer (CC) in female remains the main cause of deaths [1–3]. It is expanding rapidly with many new cases of CC diagnosed every year [4,5]. The treatment for CC includes radiotherapy, chemotherapy, and surgery, which have limited effect due to difficulties for perdition [6,7]. For females carrying HPV, CC remains to be a high risk of morbidity and mortality, and negatively affects the life quality of patients [8,9]. Therefore, more effective therapeutic targets should be developed to improve the treatment of CC.

Long non-coding RNAs (lncRNAs) can function as competing endogenous RNAs, as well as the sponges for microRNAs (miRNAs) [10,11]. LncRNAs can act as cancer prognosis indicators due to their critical roles in tumor cell progression or cell apoptosis in different human cancers by regulating the expression of mRNA [12,13]. In recent years, several lncRNAs have been reported to be involved in CC, such as lncRNA-p21 [14] and HOXA11-AS [15]. ZFAS1 was identified as a novel lncRNA that has potential functions in human cancer pathological and physiological processes [16,17]. Dysregulation of ZFAS1 has been found in hepatocellular carcinoma, gastric, colorectal, breast cancer and acute myocardial infarction patients [18–21]. Accumulating evidence has suggested that ZFAS1 participated in tumor initiation and progression. And studies have shown that the expression of ZFAS1 was correlated with chemosensitivity and prognosis of CC [22]. It was reported that ZFAS1 increased CC tumor growth and cell proliferation by upregulating LIN28 [23]. In addition, ZFAS1 enhanced CC cell metastasis by sponging miR-647 [24]. These findings suggest that ZFAS1 participated in CC progression.

Increasing evidence has shown that the interactions between miRNAs and lncRNAs could affect their mutual expression in human cancers. MiRNAs regulate gene expression through mRNA degradation or translational inhibition [25] in various human cancers [26,27]. MiR-190a is located in the tail intron regions of two genes on 15q22.2 [28]. It was reported that that the expression levels of miR-190a-5p are reduced in CC cells [29]. However, the role of miR-190a-3p in CC and the correlation between miR-190a-3p and ZFAS1 in CC were unclear.

MiRNAs may regulate cell biological activities by targeting mRNA [30]. Kruppel-like factors (KLFs) are related to many biological processes, such as carcinogenesis [31]. Our preliminary bioinformatic analysis using Targetscan revealed that KLF6 was a potential target of miR-190a-3p. KLF6 was reported to be a suppressor gene of tumor in prostate cancer [32], colorectal cancer [33], and hepatocellular carcinoma [34]. Its functional suppression of cell proliferation could inhibit cancer cell migration, and mutation, proliferation and tumor growth. However, some studies reported that KLF6 was upregulated in CC and was closely involved in CC cell growth [35,36]. Therefore, this study focused on the mechanism of KLF6 in the promotion of CC.

The lncRNA-miRNA-mRNA network plays a vital role in various human cancers [37–39]. Therefore, we hypothesized that the ZFAS1/miR-190a-3p/KLF6 axis might be involved in CC progression. Here, this study was carried out to investigate the roles and associations of ZFAS1, miR-190a-3p, and KLF6 in CC cell proliferation, and the enhancement of cervical tumor growth. Our results revealed altered expression of ZFAS1 in CC. Furthermore, we identified that miR-190a-3p was a target of ZFAS1 with two specific binding sites. Our findings would provide valuable insights into the application of ZFAS1 as a target therapy and prognosis factor.

## Materials and methods

### Samples

CC and the adjacent normal tissues from female CC patients (n = 53, 35 to 58 years old with a mean age of 48 ± 3.6 years old) who underwent surgeries between 2014 and 2018 at the First Affiliated Hospital of Zhengzhou University were collected. Patients did not receive radiotherapy or chemotherapy prior to surgeries. The Ethics Committee of the aforementioned hospital approved this study. All patients signed the informed consent. The clinicopathologic parameters of the 53 CC patients were shown in Table 1.

**Table 1.** Association of ZFAS1 and miR-190a expression with clinicopathological characteristics of patients with cervical cancer

clinicopathological characteristics	ZFAS1 expression			miR-190a expression		
	low (n = 27)	high (n = 26)	P-value	low (n = 27)	high (n = 26)	P-value
Years			P > 0.05			P > 0.05
≤48	15	10		11	15	
>48	12	16		16	11	
histological grading			P > 0.05			P > 0.05
Well and middle	16	9		10	16	
Low	11	17		17	10	
FIGO staging			**P < 0.01**			**P < 0.01**
I–II	17	7		10	18	
III–IV	10	19		17	8	
Tumor diameter			**P < 0.01**			P > 0.05
≤4 cm	23	15		16	12	
>4 cm	4	11		11	14	
Lymphnode metastasis			P > 0.05			P > 0.05
positive	17	17		9	4	
negative	10	9		18	22	

### Cell culture

293 T, SiHa and CaSki cells were obtained from Chinese Academy of Sciences and cultured in DMEM with 100 μg/ml phytomycin, 100 U/ml penicillin, and 10% FBS (Gibico, USA) with 5% CO_2_ at 37℃. MiR-190a-3p inhibitors, shZFAS1, and control were purchased from GenePharma, China. SiRNA-KLF6 was purchased from (Ribobio, China). siRNA sequences were: KLF6 antisense, 5’-UUGUAACAAAAGCUCGGGCtg-3’ and sense, 5’-GCCCGAGCUUUUGUUACAAtt-3’. The Lipofectamine 2000 reagent (Invitrogen, USA) was used for transfection.

### Quantitative real-time PCR

Total RNAs were extracted from cultured cells or brain tissues using TRIzol reagent as previously described [22]. Total RNAs (1 μg) were then reverse transcribed into cDNA. qPCR was performed using SYBR® Green Master Mix. GAPDH and U6 were used as the internal controls. The 2^–ΔΔCT^ method was used to calculate the relative gene expression levels. The primers sequences were:

ZFAS1, 5’-ACGTGCAGACATCTACAACCT-3’ (forward) and 5’-TACTTCCAACACCCGCAT-3’ (reverse); miR-190a-3p, 5’-GCAGGCCTCTGTGTGATATGT-3’ (for-ward) and 5’-GGCAAGACACTGTAGGAATATGT-3’ (reverse); KLF6, 5’-GGCAACAGACCTGCCTAGAG-3’ (forward) and 5’- CTCCCGAGCCAGAATGATTTT-3’ (reverse); U6, 5’-CTCTCTGCGGCAGCACA-3’ (forward) and 5’-AACGCTGTACGAATGTGAGT-3’ (reverse);

GAPDH, 5’-GGGAGCCAAAAGGGTCAT-3’ (forward) and 5’-GAGTCCTTCCACGATACCAA-3’ (reverse).

### Cell proliferation assay

Cell-counting kit-8 (CCK-8) assay was used to evaluate cell proliferation. Briefly, 2 × 10^3^ cells were placed into 96-well plates. When cells were adhered, CCK-8 reagent (10 μl) was added and cells were incubated for 2 h. Cell proliferation was then detected at 450 nm. Proliferation rate was measured at 1 d, 2 d, and 3 d after cell transfection.

### Colony formation

For colony formation assay, cells were plated with 200 cells/well in six-well plate. After incubation at 37° for 14 d, cell colony (> 50 cells) was measured using 0.5% crystal violet for 10 min and observed under a microscope.

### Migration and invasion assay

Using Matrigel-coated membranes, cells were seeded in the upper chamber, and the bottom chamber was filled with medium. After cells were incubated in the membrane, cells on the upper chamber were cleared away using cotton swabs. Besides, 0.1% crystal violet was used to stain cells on the bottom chamber. Then, 5 fields were randomly selected, viewed and counted.

### Dual-luciferase reporter assay

The detection method of dual luciferase reporter gene is as previously described [40]. The wild-type or mutant ZFAS1-WT was inserted into pmiR-RB-REPORT™ (Ribobio, China) to construct 2 reporter plasmids. ZFAS1 or KLF6 expression vector was constructed using pcDNA3.1 (Invitrogen) as the backbone. Negative control (NC) miRNA and miR-190a-3p mimic and inhibitor were obtained from Sigma-Aldrich, and transfected into cells. After 2 d, Dual-Luciferase assay was conducted to detect the luciferase activities.

### RIP assay

Cells lysate was cultured with IgG linked magnet bead or anti-Ago2 lined bead using Magna RNA-binding protein immunoprecipitation kit (Millipore, USA). Proteins reacted with Proteinase K and isolated immunoprecipitated RNA. RNAs were extracted and RT-PCRs were performed.

### Western blotting

Total proteins were isolated and separated on SDS-PAGE gels, followed by transferring to the membranes of PVDF (BioRad, USA), which were blocked by 5% BSA at 37℃ for 2 h. Next, the primary antibodies were used to incubate the membranes at 4℃ overnight. Then, the secondary antibody was used to incubate the membranes at 37℃ for 1 h. The signals were then detected by Odyssey infrared system.

### Lentivirus generation and establishment of the SIHa and CaSki cells with knockdown of ZFAS1

The SIHa and CaSki cells stably with the knockdown of ZFAS1 were established as previously described [41]. First, 293 T cells were seeded in 6-well plates at a density of 5 × 10^5^ cells. After transfection of shZFAS1 or control shRNA shcon, cell supernatant /PEG-*IT* mixture was centrifuged at 4°C at 1,500 × *g* for 30 min. Lentiviral particles containing shZFAS1 or shcon was used to infect SIHa and CaSki cells, which were then cultured in RPMI 1640 medium with 1 μg/ml puromycin.

### In vivo *experiment*

Female mice (4-week-old) were purchased from the Shanghai LAC Laboratory (Shanghai, China). PBS (100 μl) containing 5 × 10^6^ shZFAS1 or shcon transfected SIHa and CaSki cells were subcutaneously inoculated into mice (n = 5) at the left flank. After 35 d, mice were sacrificed. The weight and volume of tumor were measured and calculated. The Animal Experiment Ethics Committee at the First Affiliated Hospital of Zhengzhou University approved this study.

### Data analysis

Data were analyzed by SPSS 18.0 software and presented as the mean ± standard deviation (SD). *P* < 0.05 was considered as significant difference. ANOVA, or χ^2^, Student’s t-test and Pearson correlation analysis were carried accordingly. Log-rank test and Kaplan-Meier method were used for survival rate assessment.

## Results

### The expression of ZFAS1, miR-190a-3p and KLF6 in CC

**Figure 1.**
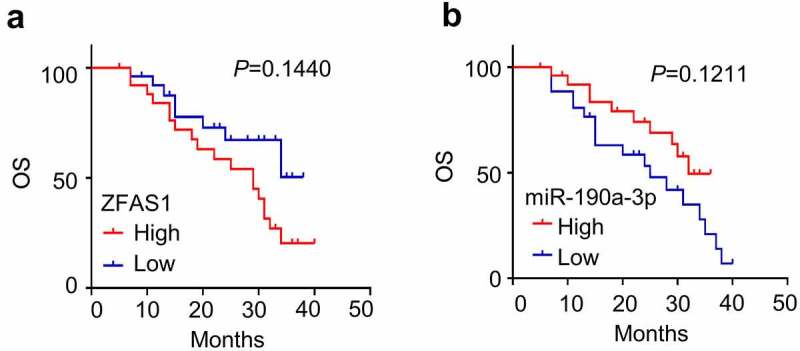
**The five year survival in CC patients**. (a)Low ZFAS1 expression group had low 5 year survival rate. (b) Low miR-190a-3p expression group had high 5 year survival rate.

Using the median expression level of ZFAS1and miR-190a-3p as the cutoff value, 53 patients were divided into low and high expression group. High ZFAS1 expression exhibited the higher overall survival rate than low expression group (Figure 1(a)) when high miR-190a-3p expression exhibited low overall survival rate (Figure 1(b)). The Chi square test revealed that ZFAS1 expression was tightly associated with tumor FIGO stage and tumor diameter (*p *< 0.01). Meanwhile, miR-190a-3p expression was also associated with tumor FIGO stage (*p *< 0.05) (Table 1).

**Figure 2.**
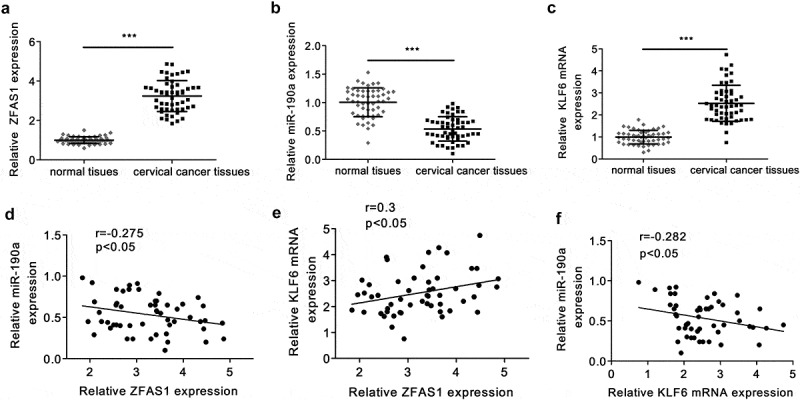
**The expression of ZFAS1, miR-190a-3p, KLF6 in CC patients**. (a) ZFAS1 expression in CC patients was detected by RT-qPCR. (b) miR-190a-3p expression in CC tissues was evaluated by RT-qPCR. (c) KLF6 expression in CC tumors. (d) The correlation between ZFAS1 and miR-190a-3p. (e) ZFAS1 was positively correlated with KLF6 in CC tumors. (f) MiR-190a-3p was inversely associated with KLF6 in CC. ***p < 0.001.

Besides, it showed that the expression levels of ZFAS1 and KLF6 were increased and the expression levels of miR-190a-3p were decreased in CC tumor tissues compared with that in normal tissues (Figure 2(a–c), *p *< 0.001). In addition, the expression of ZFAS1 was inversely correlated with the expression of miR-190a-3p, but positively correlated with the expression of KLF6 in CC tumors (Figure 2(d,e), *p *< 0.05). Moreover, the expression of miR-190a-3p and KLF6 were inversely correlated in CC tumor tissues (Figure 2(f), *p *< 0.001).

### Knockdown of ZFAS1 inhibited the function of CC cells

**Figure 3.**
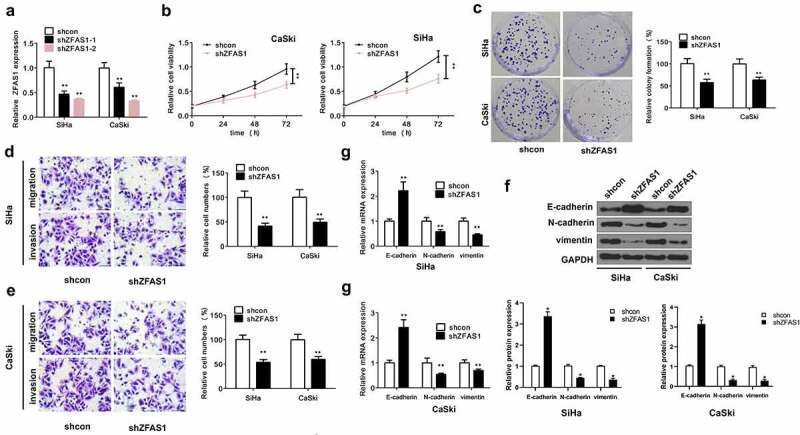
**ZFAS1 suppression inhibited CC cell function**. (a) The efficiency of shZFAS1 in CC cells. (b) ZFAS1 Knockdown inhibited cell proliferation. (c) Colony formation assay. Silencing of ZFAS1 remarkably inhibited CC cell migration (d) and invasion (e) The expression of E-cadherin, N-cadherin, and Vimentin at mRNA (g) and protein level (f) after ZFAS1 silencing. All above experiments were repeated for 3 times.

shZFAS1-1 and shZFAS1-2 were transfected into CaSki and SiHa cells. shZFAS1-2 exhibited a higher silencing efficiency than that of shZFAS1-1 (Figure 3(a), *p *< 0.01), which was then used for the subsequent experiments. In cell proliferation assay, the survival rates of SiHa and CaSki cells transfected with shZFAS1 were reduced compared with cells transfected with sh-NC (Figure 3(b), *p *< 0.01). Moreover, knockdown of ZFAS1 reduced cell colony number of both SiHa and CaSki cells (Figure 3(c), *p *< 0.01), and inhibited CC cell invasion and migration (Figure 3(d,e), *p *< 0.01). In addition, knockdown of ZFAS1 enhanced the expression levels of E-cadherin at both mRNA and protein levels, but decreased the expression levels of N-cadherin and Vimentin in CC cells (Figure 3(g,f), *p *< 0.01).

### ZFAS1 inhibited the expression of miR-190a-3p

**Figure 4.**
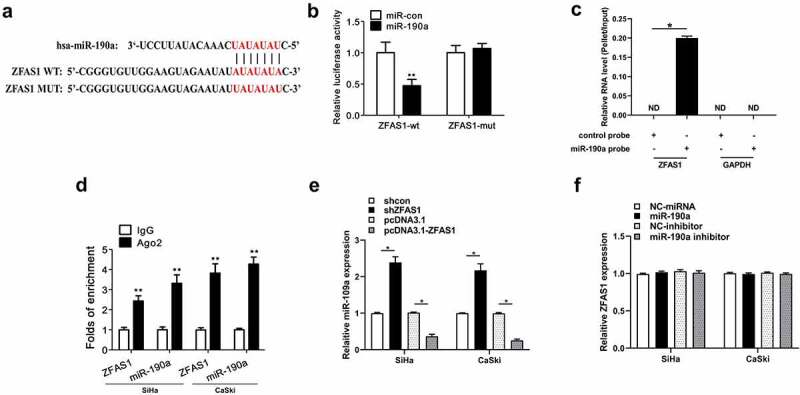
**MiR-190a-3p was a regulatory target of ZFAS1**. (a) miR-190a-3p and ZFAS1 was predicted by bioinformatics software IntaRNA 2.0. (b) Luciferase activity was conducted to further confirm this binding. The interaction between ZFAS1 and miR-190a-3p detected by RNA pull-down (c) and RIP (d). (e) miR-190a-3p expression was elevated by shZFAS1 and decreased by overexpression of ZFAS1 in CC cells. (f) Both overexpression and inhibition of miR-190a-3p did not affect ZFAS1 expression in CC cells. All above experiments were repeated for 3 times. **p < 0.01; *p < 0.05.

Due to the fact that the expression of ZFAS1 in CC cells was negatively correlated with the expression of miR-190a-3p, the correlation between ZFAS1 and miR-190a-3p was further studied. The interaction between miR-190a-3p and ZFAS1 was predicted by IntaRNA 2.0, and the results showed that they might target each other (Figure 4(a)). Luciferase activity in CC cells with the transfection of miR-190a-3p mimic plus ZFAS1-Wt was significantly lower than that with the transfection of miR-con plus ZFAS1-Mut (Figure 4(b), *p *< 0.01). The interaction between miR-190a-3p and ZFAS1 was verified (Figure 4(c), *p *< 0.05). Furthermore, both miR-190a-3p and ZFAS1 existed in the combination enriched by Ago2 antibody, but not normal IgG (Figure 4(d), *p *< 0.01). Moreover, shZFAS1 could elevate the expression levels of miR-190a-3p, and overexpression of ZFAS1 inhibited the expression of miR-190a-3p (Figure 4(e), *p *< 0.01). However, overexpression or inhibition of miR-190a-3p did not affect the expression of ZFAS1 (Figure 4(f)).

### ZFAS1 promoted the expression of KLF6 by regulating miR-190a-3p

**Figure 5.**
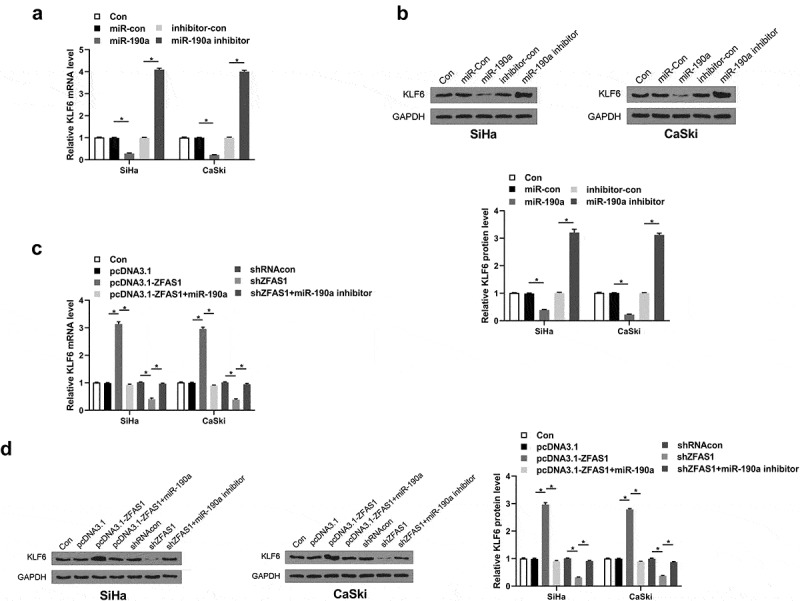
**ZFAS1 targeted miR-190a-3p to elevate KLF6 expression in CC**. (a) KLF6 expression after miR-190a-3p overexpression and/or silencing in CC cells was detected using RT-qPCR. (b) KLF6 expression in CC cells. (c) and (d) The effect of overexpression or silencing of ZFAS1 on the expression of KLF6 in CC cells. All above experiments were repeated for 3 times. *p < 0.05.

Next, the underlying mechanism of miR-190a-3p or ZFAS1 in CC was investigated. It showed that overexpression of miR-190a-3p could significantly inhibit the expression of KLF6. On the contrary, miR-190a-3p inhibitor elevated the expression of KLF6 (Figure 5(a,b), *p *< 0.05). Moreover, ZFAS1 exhibited the opposite effect on the regulation of KLF6 in CC cells (Figure 5(c,d), *p *< 0.05).

### ZFAS1 positively regulated CC cell function

**Figure 6.**
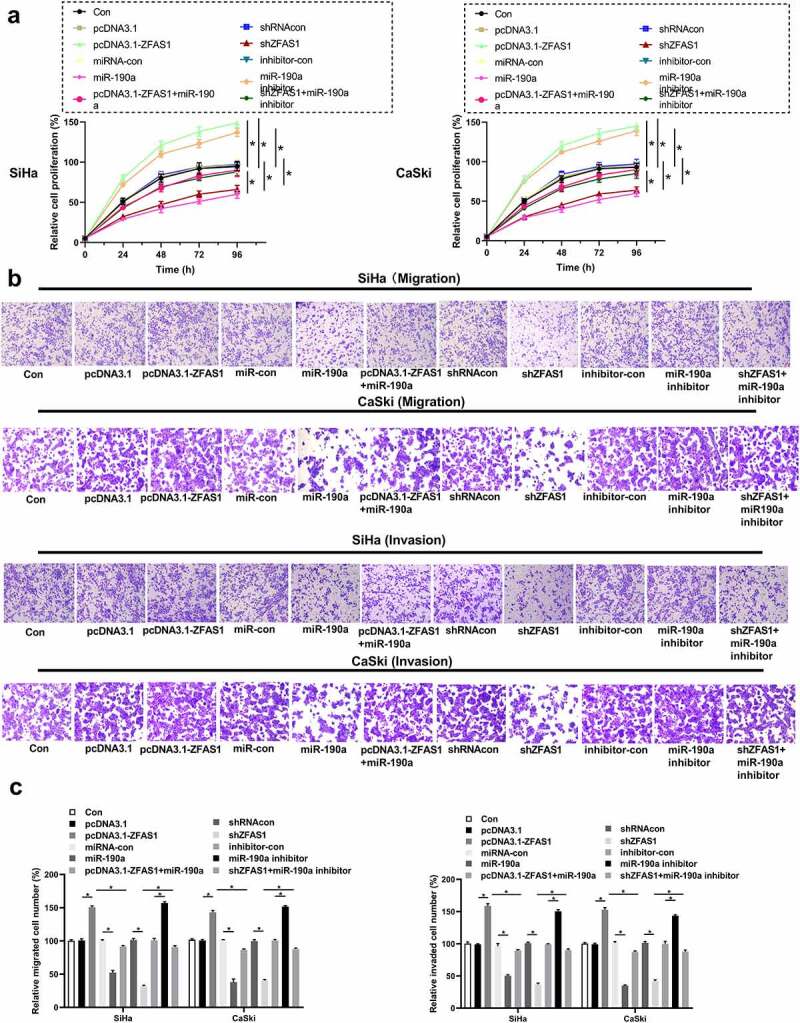
**Overexpression of ZFAS1 promoted CC cell function**. (a) Cell proliferation assay. (b) and (c) The same effects of ZFAS1 or miR-190a-3p on CC cell invasion and migration were observed in trans-well assay. All above experiments were repeated for 3 times. *p < 0.05.

It was observed that overexpression of ZFAS1 or inhibition of miR-190a-3p promoted CC cell proliferation (Figure 5(a), *p *< 0.05), while silencing of ZFAS1 or overexpression of miR-190a-3p significantly inhibited CC cell proliferation (Figure 6(a), *p* < 0.05). In addition, overexpression of KLF6 promoted and knockdown of KLF6 inhibited CC cell proliferation (Figure 1, *p* < 0.05). Moreover, miR-190a-3p inhibitor could abolish CC cell proliferation mediated by knockdown of ZFAS1 (Figure 6(a), *p *< 0.05). Furthermore, overexpression of ZFAS1 and inhibition of miR-190a-3p increased the migrated or invaded cell number (Figure 6(b,c), *p* < 0.05).

### *Knockdown of ZFAS1 promoted cervical tumor growth* in vivo

**Figure 7.**
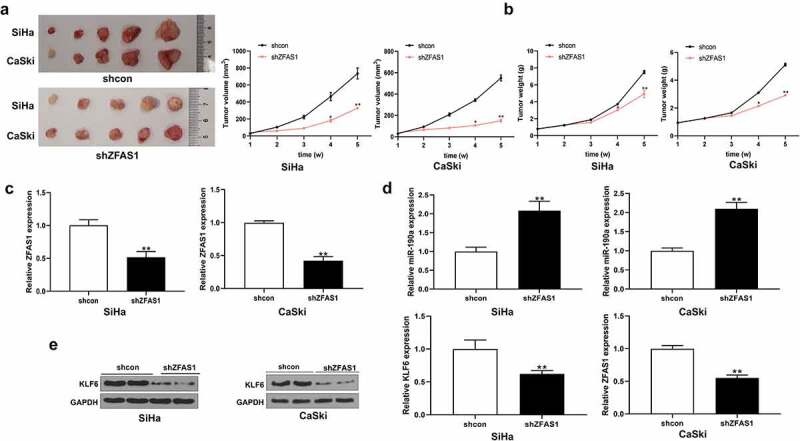
**Silencing of ZFAS1 inhibited CC tumor growth *in vivo.*** (a) and (b) silencing of ZFAS1 reduced CC tumor volume and tumor weight. (c) The silencing effect of shZFAS1 on the expression of ZFAS1 in CC tumors. (d) MiR-190a-3p expression in CC tumors transfected by shZFAS1. Obviously, shZFAS1 significantly elevated miR-190a-3p expression in CC tumors. (e) Silencing of ZFAS1 suppressed the expression of KLF6 in CC tumors. All above experiments were repeated for 3 times. **p < 0.01.

*In vitro* experiments showed that ZFAS1 promoted the expression of KLF6 by regulating miR-190a-3p, thereby affecting the function of CC cells. In our *in vivo* experiment, knockdown of ZFAS1 significantly decreased CC tumor volume generated by SiHa cell or CaSki cells (Figure 7(a), *p *< 0.01). Also, there was a significant reduction in tumor weight after mice were transplanted with CC cells transfected with shZFAS1 (Figure 7(b), *p *< 0.01). After CC tumors were formed in mice, knockdown of ZFAS1 was confirmed (Figure 7(c), *p *< 0.01). In addition, it was observed that knockdown of ZFAS1 significantly increased the expression levels of miR-190a-3p in CC cells (Figure 7(d), *p *< 0.01). However, shZFAS1 could remarkably inhibit the expression of KLF6 in CC tumors (Figure 7(e), *p *< 0.01). These results suggested that ZFAS1 was a positive factor for the regulation of CC tumor formation and growth.

## Discussion

CC ranges the second most severe cancer for females all over the world. Despite advances and developments in treatments such as radiotherapies and chemotherapies, it still remains to be one of the highest risks for cancer-related deaths in HPV infected women. Therefore, it is quite urgent to develop more accurate methods for effective treatment and prognosis for patients diagnosed with CC. LncRNAs and miRNAs are two groups of ncRNAs that participate in the regulation of various diseases. Exploration of their associations and functional mechanisms can facilitate the development of new therapeutic approaches for human cancers.

Studies have demonstrated that the progression of CC involves in the dysregulation of miRNAs, lncRNAs, and related mRNAs and proteins [42]. Previous studies revealed that ZFAS1 participated in gastric cancer cell function [43]. In addition, silencing of ZFAS1 could induce the apoptosis of gastric cancer cells [43]. It was reported that ZFAS1 could regulate the expression of Sp1 in ovarian cancer cell malignancy by targeting miR-150-5p [44]. ZFAS1 was shown to regulate colorectal cancer progression by interacting with the miR-150-5p/VEGFA axis [45]. Here, we found that the expression levels of ZFAS1 and KLF6 were elevated, while the expression of miR-190a-3p was inhibited in CC tumor tissues. Moreover, the expression of ZFAS1 was inversely associated with miR-190a-3p and positively correlated with the expression of KLF6, while the expression of miR-190a-3p and KLF6 were negatively correlated in CC tissues. Our results with the knockdown of ZFAS1 further confirmed this. We observed that CC cell viability, colony formation, invasion and migration were all suppressed after cells were transfected with shZFAS1, indicating that knockdown of ZFAS1 could inhibit CC cell progression. Therefore, our findings further established the role of ZFAS1 in the pathogenesis of CC. Our study for the first time reported the regulatory effect between ZFAS1 and miR-190a-3p, and ZFAS1 might serve as a miR-137 sponge in CC. It was reported that MIR31HG was a target of miR-193b [46]. However, in our study, miR-190a-3p did not affect the expression of ZFAS1, suggesting that ZFAS1 may not be a target of miR-190a-3p.

KLF6 was related to many biological pathways as a tumor-suppressive gene in various types of cancer [47,48]. However, some studies reported that KLF6 was upregulated in CC and closely involved in CC cell growth [35,36]. In our study, we revealed the mechanism of KLF6 in the promotion of CC. ZFAS1 was upregulated in CC and ZFAS1 could promote CC cell invasion, migration and proliferation via the miR-190a-3p. Moreover, the expression of KLF6 was inhibited after overexpression of miR-190a-3p at both mRNA and protein levels. Knockdown of ZFAS1 could also repress the expression of KLF6, which could be abolished by miR-190a-3p inhibitors. In addition, the effects of knockdown of ZFAS1 on CC cell invasion and migration was attenuated by miR-190a-3p-inhibitor. And we also found that KLF6 was involved in CC cell proliferation. These results indicated that ZFAS1 could regulate CC progression via the miR-190a-3p/KLF6 axis. In the mechanism study of ZFAS1 in CC, Meng *et al*. reported that ZFAS1 promoted tumor growth in CC by upregulating LIN28 [23]. Another study showed that ZFAS1 targets miR-647 to promote CC cell proliferation [24]. Similarly, we also revealed that ZFAS1 was a oncogenic gene in CC. We for the first time reported the sponge role of ZFAS1 in CC. ZFAS1 could act as the sponge of miR-190a-3p to inhibit the expression of KLF6 by reducing competitively binding to miR-190a-3p, thereby promoting CC progression. Moreover, studies revealed that ZFAS1 could promote the tumor growth of hepatocellular carcinoma [21] and gastric cancer [43] *in vivo*. For instance, it was demonstrated that overexpression of ZFAS1 could enhance gastric cancer cells tumor growth *in vivo* [43]. Our *in vivo* experiments showed that silencing of ZFAS1 reduced CC tumor size. In addition, knockdown of ZFAS1 could also suppress the expression of KLF6, and elevated the expression of miR-190a-3p, suggesting that ZFAS1 was involved in CC tumorigenesis via the miR-190a-3p/KLF6 axis. ZFAS1 may regulate cell proliferation and invasion in CC by via the miR-190a-3p/KLF6 axis. However, the exact relationship ZFAS1 and CC needs to be further investigated.

## Conclusions

ZFAS1 acted as an important regulatory factor in the development of CC via the miR-190a-3p/KLF6 axis. It might provide valuable insights into the therapy of CC.

## Supplementary Material

Supplemental Material

## Data Availability

The datasets used and/or analyzed during the current study are available from the corresponding author on reasonable request.
