# Supplementary figures and images for: Long non-coding RNA ZFAS1 regulates cell proliferation and invasion in cervical cancer via the miR-190a-3p/KLF6 axis
Source: Bioengineered. 2022 Feb 3;13(2):3840–51. doi: 10.1080/21655979.2021.2022265 (PMC8973928; doi:10.1080/21655979.2021.2022265)

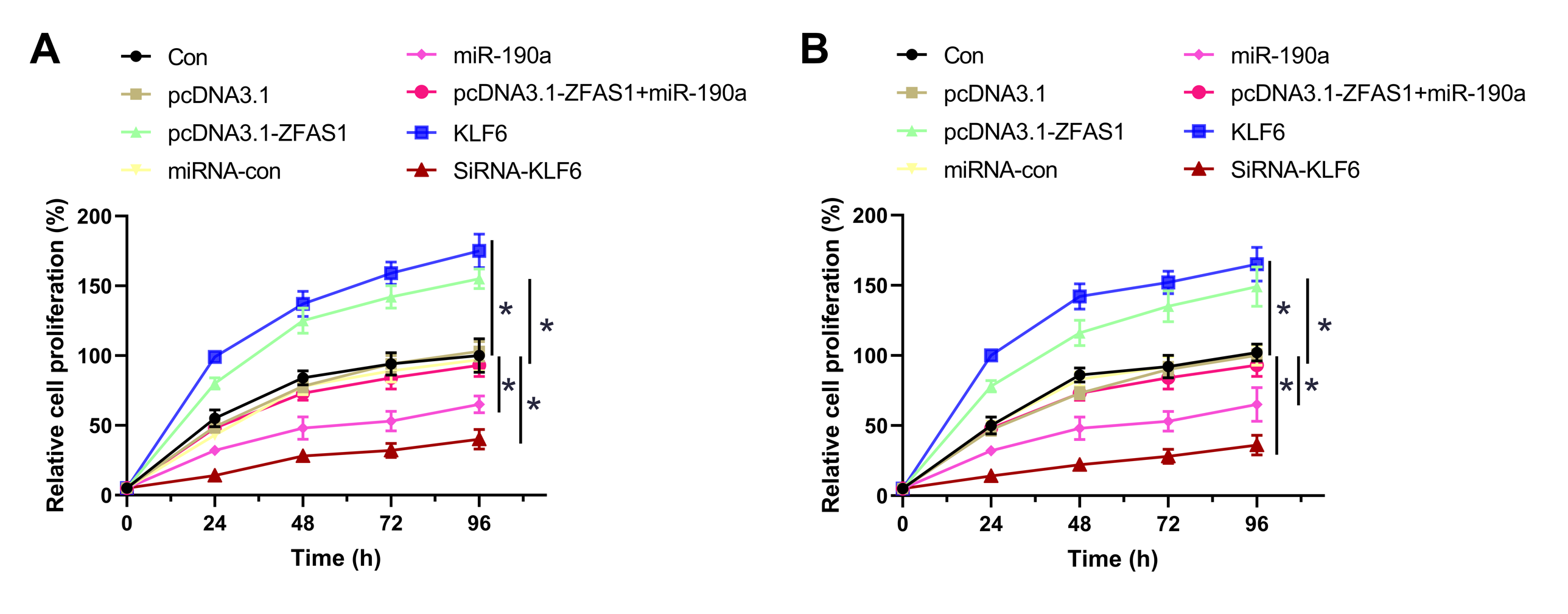

Supplement: Supplemental Material [file KBIE_A_2022265_SM8415.zip › supplementary/FigureS1 revised (1).tif]
